# Centrally Acting Skeletal Muscle Relaxants Sharing Molecular Targets with Drugs for Neuropathic Pain Management

**DOI:** 10.3390/brainsci16010067

**Published:** 2025-12-31

**Authors:** Judit Mária Kirchlechner-Farkas, David Arpad Karadi, Imre Boldizsár, Nariman Essmat, Anna Rita Galambos, Zoltán Patrik Lincmajer, Sarah Kadhim Abbood, Kornél Király, Éva Szökő, Tamás Tábi, Mahmoud Al-Khrasani

**Affiliations:** 1 Department of Pharmacology and Pharmacotherapy, Faculty of Medicine, Semmelweis University, Nagyvárad tér 4, H-1085 Budapest, Hungary; kirchlechner.farkas.judit.mari@semmelweis.hu (J.M.K.-F.); boldizsar.imre2@semmelweis.hu (I.B.J.); galambos.anna@phd.semmelweis.hu (A.R.G.); abbood.sarah@phd.semmelweis.hu (S.K.A.); kiraly.kornel@semmelweis.hu (K.K.); 2 Center for Pharmacology and Drug Research & Development, Semmelweis University, Ülloi út 26., H-1085 Budapest, Hungary; lincmajer.zoltan.patrik@semmelweis.hu (Z.P.L.); szoko.eva@semmelweis.hu (É.S.); tabi.tamas@semmelweis.hu (T.T.); 3 Department of Anesthesiology and Intensive Therapy, Semmelweis University, Üllöi út 78b, H-1082 Budapest, Hungary; karadi.david.arpad@semmelweis.hu; 4 Department of Pharmacology and Toxicology, Faculty of Pharmacy, Zagazig University, Zagazig 44519, Egypt; nariman@zu.edu.eg; 5 Department of Pharmacodynamics, Semmelweis University, Nagyvárad tér 4, H-1089 Budapest, Hungary

**Keywords:** neuropathic pain, centrally acting skeletal muscle relaxants, VGSCs, VGCCs

## Abstract

Treatment of neuropathic pain (NP) remains a challenge in clinical practice because the current treatment approaches produce satisfactory pain alleviation in only 30% of patients. This necessitates developing novel drugs or repurposing existing medications intended to manage other diseases. When the repurposing intendance is chosen, similarity in the pharmacological properties should be hosted by the candidate drugs. Herein, this review sheds light on the mechanisms of certain centrally acting skeletal muscle relaxants (CMRs), specifically tolperisone. So far, data indicate that tolperisone displays voltage-gated sodium channel (VGSC) blocking properties with modulatory effect on voltage-gated calcium channels (VGCCs). These properties have led to recent preclinical research initiatives testing tolperisone in NP, resulting in positive outcomes. Furthermore, the review highlights the currently available VGSC blockers and proposes a strategy based on combining them with VGCC blockers that have been proven for the treatment of NP. This proposal is supported by the fact that tolperisone, in combination with pregabalin, has recently been shown to acutely halt NP.

## 1. Introduction

Neuropathic pain (NP) is a complex and challenging condition and is a consequence of disease or lesion affecting the somatosensory nervous system according to International Association for the Study of Pain. Current drug discovery and repurposing strategies primarily focus on identifying compounds that interact with molecular targets that contribute to the development of NP.

Centrally acting skeletal muscle relaxants (CMRs), including baclofen, carisoprodol, cyclobenzaprine, diazepam, metaxalone, methocarbamol, orphenadrine, chlorzoxazone, tizanidine, and tolperisone, have been approved for the treatment of spastic conditions related to muscle damage and inflammation, such as acute low back pain, and some of them have shown varying levels of efficacy in reducing pain [1,2,3,4,5,6,7,8,9,10,11]. A population-based cross-sectional prevalence study was conducted in the US between 1988 and 1994 to assess CMRs’ use in the population [12]. This study reports on an estimated amount of 2 million adults using CMRs in the USA. Several studies have reported that the incidence of NP and spasticity reaches 50–60% and 70%, respectively, in patients with spinal cord injury (SCI) [13]. The current pharmacological approach for the treatment of individuals with SCI includes baclofen, tizanidine, benzodiazepines, clonidine, and gabapentinoids [14,15].

A recent study has evaluated the effectiveness of CMRs including baclofen, tizanidine, cyclobenzaprine, eperisone, quinine, carisoprodol, orphenadrine, chlormezanone, and methocarbamol in different pain types [3]. The outcome of the study indicates that baclofen, tizanidine, and cyclobenzaprine display strong evidence of effectiveness against trigeminal neuralgia (TGN), neck pain, and painful cramps [3]. On the other hand, the study has pointed out that in the context of fibromyalgia, low back pain, and other syndromes, these drugs are not more beneficial than placebo. Finally, the study has proposed that clinicians need to be attentive to potential adverse effects and should consider discontinuing medications if adequate pain management are not achieved. In this study, tolperisone was not included; it is a CMR that was first introduced by the Hungarian company Gedeon Richter Inc. in 1959. The clinical value of tolperisone as a muscle relaxant is underscored by its efficacy in treating muscle spasms while devoid of sedative effect associated with the use of other CMRs, as confirmed by numerous placebo-controlled double-blind studies [11,16,17,18]. Tolperisone thus quickly became a very popular CMR in Eastern Europe and countries of the former Soviet Union and Asia, especially in Japan.

The current treatment for the management of TGN includes first line medications such as carbamazepine and oxcarbazepine with strong recommendation; alternative or adjuvant medications include gabapentinoids, lamotrigine and baclofen with weak recommendation [19,20]. In general, in NP, the current pharmacological approaches encompass antidepressants, that modulate monoaminergic transmission, and drugs acting on ion channels as antiepileptics, including gabapentinoids, local anesthetics, capsaicin, and opioid analgesics, which point to the complex molecular background of neuropathy [21,22,23,24,25]. Carbamazepine and oxcarbazepine are considered as therapy for NP according to some studies; however, inconclusive results and patient intolerability that hinders dose escalation to achieve adequate treatment, due to a higher percentage of discontinuations resulting from various side effects, have been reported [25,26]. In fact, if we consider the mechanistic targets of the current medications used to manage NP, the gamma-aminobutyric acid B receptor (GABA_B_R) is not included.

The above-mentioned CMRs for controlling muscle spasticity and related pain have different molecular mechanisms, but some of them, cyclobenzaprine, tizanidine, and tolperisone, share molecular mechanisms with the currently available drugs for the management of NP (Table 1). Both spasticity and NP have similarities in their molecular backgrounds regarding disinhibition caused by dysfunction of descending pathways or interneurons in the spinal cord, hyperexcitability of excitatory neurons, sprouting, upregulation of excitatory receptors, glial activation, and neuroinflammation [13,27,28]. The symptoms of neuropathy, including allodynia, hyperalgesia, and paresthesia (abnormal sensations without any stimuli, e.g., burning, tickling or tingling) [29], are not adequately controlled by the current medications [30]. For details of the most commonly used CMRs and drugs for NP, refer to Table 1.

The focus of the present review is to discuss the pharmacodynamic properties of CMRs including cyclobenzaprine, tizanidine, and tolperisone that share targets with current NP medications, specifically affecting serotonergic and/or noradrenergic neurotransmission or inhibiting either voltage-gated sodium channels (VGSCs), voltage-gated calcium channels (VGCCs), or both. The review also pays attention to tolperisone per se or in combination with gabapentinoids in the context of NP, which, to the best of our knowledge, has not been reviewed thus far.

The outcome of this review may create a mechanistic rationale for the possible repurposing of the reviewed CMRs as therapeutic options for NP. Furthermore, this review brings overview on preclinical and clinical studies on these compounds in relation to analgesia that were conducted so far. Side effects and their mechanisms are also addressed as major limitations of CMR use or repurposing. Based on the presented literature data, we aim to propose further possible research in hope of translating them to clinical use.

## 2. Centrally Acting Skeletal Muscle Relaxants Sharing Molecular Targets with Current Neuropathic Pain Medications

### 2.1. Cyclobenzaprine

**Figure 1:**
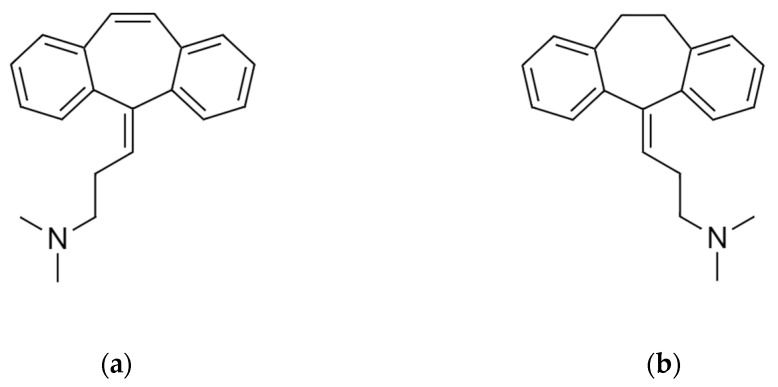
The molecular structure of cyclobenzaprine (**a**) and amitriptyline (**b**). The figures were hand drawn in an art program (Clip Studio Paint), based on the 2D structure images presented on their respective PubChem pages. Cyclobenzaprine: PubChem Identifier: CID 2895, URL: https://pubchem.ncbi.nlm.nih.gov/compound/2895#section=2D-Structure (accessed on 15 December 2025). Amitriptyline: PubChem Identifier: CID 2160, URL: https://pubchem.ncbi.nlm.nih.gov/compound/2160#section=2D-Structure (accessed on 15 December 2025).

Cyclobenzaprine has long been described to have potent muscle relaxant effects following per os administration in animal models [32]. In this regard, its pharmacological effect was proposed to affect descending noradrenergic and serotonergic pathways among others in the central nervous system (CNS) [36]. Structurally, it displays a tricyclic structure closely related to that of tricyclic antidepressants (TCAs) (Figure 1). Before the discovery of selective serotonin reuptake inhibitor antidepressants, TCAs were the gold standard pharmacological treatment for depression, and they are still important drugs for the treatment of various other conditions related to chronic pain [24]. The close structural similarity between amitriptyline and cyclobenzaprine (Figure 1) has urged research groups to investigate its mechanism of action and efficacy in analgesic tests.

Mechanistically, cyclobenzaprine was initially reported to possess α_2_-adrenoceptor (AR)-mediated agonist characteristics [36,67]. Comissiong et al. also demonstrated an increase in noradrenaline metabolism in response to cyclobenzaprine treatment in the ventral horn of the spinal cord that receives dense innervation from the locus coeruleus, as opposed to no difference observed in the zona intermedia receiving no such innervation. These results indicate that cyclobenzaprine acts as an indirect inhibitor of α-motoneuron activity by increasing the noradrenergic activity in the spinal cord that originates from the locus coeruleus [68]. However, it was unclear how this increase in noradrenaline release attenuates the action of spinal motor neurons, as most studies conducted since show an α1-AR mediated facilitation of ventral horn motoneurons in response to noradrenaline [67,69]. On the other hand, it has also been shown that electrical stimulation of the locus coeruleus induces a biphasic response in the ventral horn, which consists of facilitation, followed by inhibition. The inhibition phase was naloxone-reversible, suggesting it to be an opioid-cotransmitter mediated action [70].

Later studies have underlined the principal role of the noradrenergic system in the modulation of spinal motor neuronal networks under physiological or pathophysiological conditions. In this context, α_1_-, α_2_- and β-ARs have been found to be involved in the modulation of the function of motoneurons [71]. α_1_-ARs have been reported to directly facilitate motoneuron excitability whereas α_2_-ARs decrease it [72]. In addition, after spinal nerve injury, α_1_-ARs become constitutively active, which contributes to motoneuronal hyperexcitability and concomitant spasms. On the other hand, activation of α_2_-ARs can inhibit sensory synaptic transmission to the motoneurons, thus decreasing excitatory postsynaptic potential [72]. As aforementioned, cyclobenzaprine does increase the level of noradrenaline in the spinal ventral horn through the activation of locus coeruleus neurons and by mechanisms that have not been fully elucidated. Kobayashi and colleagues have also investigated the actions of cyclobenzaprine and have reported another possible player in its mechanism of action [36]. Their study indicates that destroying noradrenergic neurons by pretreatment with 6-hydroxydopamine did not inhibit the action of cyclobenzaprine on the monosynaptic reflex; however, inducing serotonin depletion by inhibiting its synthesis with DL-p-chlorophenylalanine significantly blocked the actions of cyclobenzaprine. Furthermore, in their study, cyclobenzaprine also attenuated the action of the 5-HT_2A_ receptor agonist, 2,5-dimethoxy-4-iodoamphetamine (DOI), belonging to psychedelic agents, which can induce both structural and functional neuroplasticity after acute administration [73]. A recent study by the Riegel research group has shown that inhibition of 5-HT_2A_ receptor in the prefrontal cortex attenuates NP developed in rats with allodynia evoked by spared nerve injury (SNI) [74].

Taken together, results indicate that besides altering the descending noradrenergic tone in the spinal cord, cyclobenzaprine also blocks α-motorneuronal excitation by acting on descending serotonergic neurons and 5-HT_2A_ receptor [36]. A number of 5-HT receptor subtypes are expressed on the membrane of motoneurons such as 5-HT_1A_, 5-HT_1B_, 5-HT_1D_, 5-HT_2A_, 5-HT_2B_, 5-HT_2C_, and 5-HT_5A_ [75,76]. The activation of an individual 5-HT receptor can induce either excitation or inhibition of the motoneurons [75]. With respect to 5-HT_2A_ receptor subtype, its activation leads to spinal motoneuron excitation [77]. Therefore, cyclobenzaprine likely blocks the effect of serotonin on motoneurons, though further studies are needed to clarify this point. Collectively, these data support that both serotonergic and noradrenergic receptors contribute to the effects of cyclobenzaprine.

Descending noradrenergic and serotoninergic pathways are important in controlling pain transmission as well. Growing data suggest the concept that chronic pain is associated with dysregulation in this pain modulatory pathway. Serotonin can produce either inhibitory or stimulatory effect, depending on the 5-HT receptor subtype undergoing activation. Thus, activation of the 5-HT_1A_, 5-HT_1B_, 5-HT_1D_, and 5-HT_7_ receptors mediates antinociception, whereas the 5-HT_2A_ and 5-HT_3_ receptors can promote nociception [78,79,80]. The presence of the 5-HT_2A_ receptor has been reported in CGRP and substance P synthesizing nociceptive neurons [81,82]. The exact role and exploitability of the 5-HT_2A_ receptor signaling in chronic pain management models is highly controversial. With respect to NP, the picture is even more complicated with contradictory results. On one hand, 5-HT_2A_ antagonists, ketanserin and sarpogrelate, alleviated neuropathic symptoms induced by sciatic nerve ligation (SNL) or chronic constriction injury (CCI) and, more recently, intrathecally administered α-phenyl-1-(2-phenylethyl)-4-piperidinemethanol, another 5-HT_2A_ receptor antagonist, was shown to be effective in 2′,3′-dideoxycytidine-induced peripheral neuropathy (PN) [83,84,85]. On the other hand, evidence supports that 5-HT_2A_ receptor activation largely participates in the alleviation of allodynia in rats [86]. Elucidating the cause of data discrepancy requires further investigation.

Cyclobenzaprine has also been reported to increase the spinal serotoninergic tone and can cause serotonin syndrome as a side effect [87,88,89]. Thus, beside the action of cyclobenzaprine on the 5HT_2A_ receptor, it also displays serotonin enhancer effects, which might stem from the inhibition of the reuptake of serotonin [88]. Consequently, the increased serotonin level in the spinal cord could also activate 5-HT receptors that tend to inhibit pain transmission.

At clinical level, two small studies were also conducted in order to assess the efficacy of cyclobenzaprine in myofascial pain. In one study, patients receiving cyclobenzaprine reported statistically significant improvement in symptoms compared to clonazepam or placebo [90]. In the second study, cyclobenzaprine proved to have statistically equivalent efficacy with infiltered lidocaine [91]; however, the route of administration was different, and the authors suggested that the analgesic effect of infiltrated lidocaine was superior. On the other hand, oral cyclobenzaprine was more favorable in terms of patients’ comfort and compliance because of the invasiveness of lidocaine administration [92].

The above-mentioned effect of cyclobenzaprine on the spinal monoaminergic system and its structural similarity to amitriptyline could suggest exploitable analgesic properties, though no clinical studies have been carried out to evaluate the analgesic effect of cyclobenzaprine alone or in combination with analgesics following either short- or long-term treatment in NP conditions. Nevertheless, some studies proposed treatment combinations involving cyclobenzaprine and non-steroidal anti-inflammatory drugs (NSAIDs) for back pain or painful muscle spasms [5,93]. NSAIDs are well known to produce both peripheral and central analgesic effects by inhibiting the cyclooxygenase enzymes and, consequently, the prostaglandin and other inflammatory mediator synthesis that might contribute to the peripheral sensitization of nociceptors [94]. Indeed, in clinical studies cyclobenzaprine is combined with NSAIDs to achieve analgesic effect in acute back pain; however, NSAIDs are not included in the current treatment approach of NP. In addition, cyclobenzaprine is not included in the current pharmacological approach for the treatment of individuals with SCI. Thus, novel combinations of cyclobenzaprine and compounds used in neuropathic states could be worth investigating. This is because some studies suggest that using combination-based therapy may enhance analgesic efficacy and reduce side effects.

The impact of cyclobenzaprine on VGSCs was only suggested once in the context of cardiac sodium channel blockade through a case report of ventricular tachycardia and related to its unwanted effects [95]. To the best of our knowledge, there is no study focusing on the effect of cyclobenzaprine on neuronal sodium channels, which are potential targets for the treatment of chronic pain conditions. A recent comprehensive pharmacovigilance analysis has revealed that adverse events associated with cyclobenzaprine may pose potential risks, including toxicity and suicidal behavior [96]. Further evidence of important, rare but serious adverse effects of cyclobenzaprine, including serotonin syndrome (particularly when used in combination with other serotoninergic agents) or prolongation of conduction time, arrhythmias, and tachycardia, similarly to TCAs, has also been reported [33,35,89].

### 2.2. Tizanidine

Tizanidine was first described in 1980 [37,38], and is a potent central α_2_-AR agonist with muscle relaxant properties. Structurally, tizanidine is related to clonidine, yet its antispastic efficacy is similar to that produced by baclofen while having a more favorable side effect profile [41]. In the context of analgesia, several preclinical and clinical studies have shown that tizanidine produces significant antinociceptive, anti-thermal hyperalgesic and antiallodynic effects that are mediated by α_2_-ARs [39,97,98,99,100,101,102]. An early autoradiographic localization study, along with others, found high densities of α_2_-ARs in the substantia gelatinosa of the spinal cord, the spinal trigeminal nucleus, and the locus coeruleus, indicating the involvement of α_2_-ARs in pain regulation [103,104]. There is also evidence on α_2_-ARs mediating analgesia and sedation at different sites in the CNS. Buerkle et al. found that activating spinal α_2_-ARs mainly causes analgesia with limited sedation, while activating α_2_-ARs in the brain induces strong sedation with little antinociceptive action [105]. α_2_-ARs can be categorized as α_2A_-AR, α_2B_-AR, and α_2C_-AR [106,107,108] and their effects can be selectively antagonized by BRL-44408, imiloxan, and JP-1302, respectively [109,110,111]. Several studies have been carried out with the aim of elucidating the neuroanatomical distribution of α_2_-ARs. An in situ hybridization study by Nicholas et al. found the mRNA of the α_2A_-AR subtype to be most prevalent in the locus coeruleus, ventrolateral medullary reticular formation, and the intermediolateral cell column of the thoracic spinal cord, among others. On the other hand, high levels of the α_2C_-AR subtype mRNA were found in the DRG [112]. Several studies have demonstrated the contribution of different α_2_-AR subtypes to the development of NP and its attenuation by α_2_-agonists [113,114]. Evidence suggests that α_2_-ARs play a critical role in modulating pain perception. α_2_-ARs activation leads to significant changes in synaptic transmission within the dorsal part of the spinal cord. Notably, this activation results in the inhibition of VGCCs and the activation of potassium channels at presynaptic and postsynaptic membranes, respectively [113]. Studies have also pointed to α_2A_-AR subtype contribution to the antiallodynic action of α_2_-AR agonists [115,116,117]. Furthermore, a study by Pei and coworkers demonstrated that intrathecal tizanidine can produce analgesic effects against mechanical and thermal hyperalgesia in neuropathic rats, which is reversible by α_2A_-AR antagonist, BRL-44408 [98]. These results support the contribution of α_2A_-AR subtype to the analgesic effect of tizanidine. All types of α_2_-ARs are classified within the G-protein coupled receptor (GPCR) family, and primarily coupling to the Gi/Go subfamilies [118,119].

With respect to VGSCs (Nav), studies have shown the ability of α-AR agonists such as dexmedetomidine to inhibit Nav 1.7 and Nav1.8 through the activation of Gi/o α_2_-ARs on the primary sensory neurons of the trigeminal ganglia [120]. Tizanidine, at supraclinical concentrations, was found to affect the cardiac rapid delayed rectifier potassium current (IKr) associated with human ether-a-go-go-related gene (HERG) channels, leading to a lengthening of the action potential duration as measured by monophasic action potential duration at 90% repolarization (MAPD90), accompanied by QTc prolongation [121]. To the best of our knowledge, there are no details regarding the direct impact of tizanidine on VGSCs. It would be of interest to investigate tizanidine in combination with blockers of VGSCs, specifically those that inhibit subtypes of channels distributed in peripheral sensory neurons.

Finally, tizanidine is included in the current SCI treatment approach; however, future studies are needed to develop further α_2_-AR subtypes-selective agonists, which might have different pharmacological properties related to spasticity, pain, or common adverse effects such as xerostomia and drowsiness, among others [41]. In the future, examining the combination of tizanidine with VGSC or VGCC blockers, such as tolperisone or peripheral acting new Nav 1.7, 1.8 inhibitors (see below) might show therapeutic value in NP. VGSC blockers that are devoid of sedative effect might positively or neutrally affect the side effects of tizanidine, improving side effect profile while increasing the antiallodynic effect of each other in subjects with NP.

### 2.3. Tolperisone

In this section, we aim to overview the proposed mechanism of tolperisone in relation to muscle-associated pain in both clinical and preclinical scenarios, emphasizing its current and future implications for NP. As mentioned in the introduction, tolperisone is a CMR and is devoid of the sedative effect, as described in several preclinical and clinical studies, in relation to pain associated with musculoskeletal disorders [16,122].

**Figure 2:**
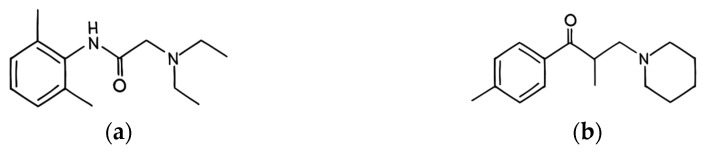
The molecular structure of lidocaine (**a**) and tolperisone (**b**). The figures were hand drawn in an art program (Clip Studio Paint), based on the 2D structure images presented on their respective PubChem pages. Lidocaine: PubChem Identifier: CID 3676, URL: https://puchem.ncbi.nlm.nih.gov/compound/3676#section=2D-Structure (accessed on 15 December 2025). Tolperisone: PubChem Identifier: CID 5511, URL: https://pubchem.ncbi.nlm.nih.gov/compound/5511#section=2D-Structure (accessed on 15 December 2025).

Structurally, tolperisone shows similarity to lidocaine, sharing the typical moieties necessary to the effect of local anesthetics; the lipophilic aromatic ring and the basic amine group tethered by a short linkage (Figure 2) [123]. Their chemical similarity raises the possibility of the interaction of tolperisone with VGSCs. In addition, based on the chemical structure, Ono and coworkers have attributed its measured muscle relaxant activity to sodium channels located on the axonal membranes similarly to local anesthetics [42]. In this context, Hinck and Koppenhöfer reported that tolperisone caused a significant depressive effect on the voltage-gated sodium currents, while only mildly affecting the potassium permeability, resulting in a slight increase in potassium currents at weak depolarization and a slight decrease at higher depolarization in Ranvier nodes of the sciatic nerve of Xenopus frogs [45]. In other research strategies that focus on the impact of tolperisone on calcium currents, Novales-Li and his coworkers, utilizing voltage clamping, have found that tolperisone does suppress calcium currents in a dose-dependent manner and shifted the steady state inactivation curves towards the hyperpolarizing direction [43]. In these experiments, tolperisone analogs eperisone and isoperisone showed a significantly more potent action (lower IC_50_ value) than tolperisone on calcium currents.

The discovery and characterization of VGSCs, specifically Nav1 (Nav1.1–Nav1.9), have opened a new area of target research related to drugs acting on sodium channels [124,125]. Nav 1 subtype channels involved in pain are summarized in Table 2. In this regard, Hofer et al. used the two-electrode voltage clamp technique to record sodium currents corresponding to different isoforms of VGSC (Nav1.2–Nav1.8) expressed in the Xenopus laevis oocyte expression system [61]. In this study, the effects of tolperisone and lidocaine were also examined. Tolperisone was found significantly more potent than lidocaine in the inhibition of sodium channel isoforms Nav1.6, Na v1.7, and Na v1.8. Their results also indicated that the extent of tonic block induced by tolperisone was significantly stronger on the isoforms Nav1.2, Nav1.3, Nav1.7, and Nav1.8 compared to lidocaine [61]. As VGSCs are recognized to have a critical role in the pathophysiology and development of neuronal hyperexcitability following peripheral nerve injury, drugs that are able to exert a high level of tonic blockade on these channels might be useful in specific clinical scenarios, for example, in chronic pain treatment [126]. Nav1.7 is expressed in DRG neurons; however, Nav1.8 exhibits selective expression within these neurons. Nav1.3, which is upregulated in DRG neurons after injury, has garnered attention from researchers and is considered a promising target for pharmacological treatment of NP [127]. Suzetrigine, the recently introduced VGSC blocking agent for clinical use, is the first oral, nonopioid small molecule with peripheral analgesic action for acute pain and demonstrates selectivity for Nav1.8 [128]. Other selective inhibitors of Nav1.7, such as aneratrigine, have been recently developed as an alternative drug of opioid analgesics [129].

The principal function of Nav1.7 and Nav1.8 in both the generation and maintenance of abnormal neuronal hyperexcitability of sensory neurons underscores the significance of these channels in the progression of pathological pain, including NP.

Owing to tolperisone’s molecular mechanisms, particularly the inhibition of both voltage-gated sodium and calcium channels, it is predicted that it may serve as a promising agent for reducing NP. Both sodium and calcium channels are crucial in transmitting pain signals within the CNS. Recent studies have highlighted that tolperisone’s ability to modulate these channels may effectively attenuate NP in rodents. In this regard, in a study conducted by Lakatos et al., oral tolperisone has shown acute antiallodynic effects in a rat model of NP, specifically examining mechanical allodynia (mechanical pressure stimulation) of rats with experiencing mononeuropathic pain induced by partial sciatic nerve ligation [141]. Besides suppressing mechanical allodynia, tolperisone has also been demonstrated to effectively attenuate tactile allodynia in the same model of NP [142]. In this context, Essmat et al. have shown that tolperisone can only exhibit antiallodynic effects against tactile allodynia following chronic treatment. These studies collectively underscore that tolperisone has therapeutic potential in mitigating symptoms associated with NP at a preclinical scenario.

VGCC (Cav) types, in particular, ones hosting α_2_δ_1_ subunit are hitherto described as the molecular target of gabapentin and pregabalin, which are first-line medications in the treatment of NP [52,143] (Table 1). The current drugs with calcium channel modulatory action that have been validated as effective against NP in both preclinical and clinical settings selectively bind to the α_2_δ-1 and α_2_δ-2 subunit containing VGCCs. The α_2_δ subunits play a crucial role in the functional assembly of VGCCs, specifically Cav2.1 and Cav2.2, which are distributed at key relay points within the pain pathways (see Table 3).

Despite the effectiveness of VGCC modulators like gabapentinoids in the management of NP, the slow onset of action has been described previously [144]. To this end, the combination-based therapies including VGSC and VGCC blockers’ combination to combat pain have been recognized as a promising strategy. In this context, a study by Hahm has shown that carbamazepine and pregabalin combination ameliorates NP of rats [145]. In a different study, the combination of carbamazepine and gabapentin resulted in better pain management for patients suffering from TGN compared to the administration of carbamazepine per se [146]. This strategy was followed by a study carried out in our previous work regarding the enhancement of tolperisone’s efficacy in NP [142]. In this regard, when tolperisone and pregabalin were administered simultaneously, a significant acute antitactile allodynic effect with fast onset was observed, reinforcing the effectiveness of combination of VGSC and VGCC blockers in managing NP [142]. The aspect of the present combination avoids concerns that have been raised regarding the combination of drugs with similar mechanisms of action, specifically the combination of TCAs with medications that positively influence serotonin levels. To the best of our knowledge, this concern is of lesser relevance in VGSC and VGCC combination, but to rule this issue out, future studies are required.

**Table 3:** The distribution of VGCC hosting α2δ1 subunit in pain pathway.

Type	Channel	Voltage Activation	Peripheral Primary Afferents/Dorsal Root Ganglia	Spinal Dorsal Horn	Thalamus	Somatosensory Cortex	References
L	Cav1.1	HVA	No	No	No	No	[147,148,149,150,151,152,153,154,155,156,157,158,159]
	Cav1.2	HVA	Yes (Low)	Yes	Yes	Yes	
	Cav1.3	HVA	No	Yes	Yes	Yes	
	Cav1.4	HVA	No	No	No	No	
P/Q	Cav2.1	HVA	Yes	Yes *	Yes	Not enough data	[157,158,159]
N	Cav2.2	HVA	Yes	Yes	No	No	[159,160,161,162,163]
R	Cav2.3	HVA	Yes	Yes	Yes	Yes (debated)	[164,165,166,167,168,169,170,171,172,173,174]
T	Cav3.1	LVA	No	Yes	Yes	Yes	[175,176,177]
	Cav3.2	LVA	Yes	Yes	No	Yes	
	Cav3.3	LVA	No	Yes (Low)	Yes	Yes	

*: Presynaptic distribution; HVA: High voltage activated; LVA: Low voltage activated.

The efficacy and safety of tolperisone have been evaluated earlier. A clinical study conducted in 2012 evaluated the efficacy of tolperisone in comparison with thiocolchicoside for alleviating acute low back pain. The study has demonstrated that tolperisone effectively reduced pain while offering a favorable safety profile, distinguishing it from other treatments [8]. In 1996, Pratzel et al. conducted a study to evaluate the effectiveness of tolperisone for treating patients with painful reflex muscle spasms, primarily associated with musculoskeletal conditions such as spondylarthrosis and intervertebral disc prolapse. Participants were randomly assigned to receive either 300 mg of tolperisone or a placebo daily for 21 days. The outcome of the study revealed that tolperisone significantly exceeded the placebo in alleviating muscle spasms [18]. Tolperisone also showed negligible adverse effects related to attention and cognitive brain functions, which make it preferable for stroke-affected elderly patients [17]. Thus, in a multicenter randomized double-blind placebo-controlled clinical study by Stamenova et al., the efficacy and safety of tolperisone were assessed in the treatment of muscle spasticity following cerebral stroke [16]. One hundred and twenty patients suffering from stroke-related spasticity (with a degree of spasticity being 2 or higher on the Ashworth Scale) were selected and randomized to either receive tolperisone or placebo for a total treatment time of 12 weeks. The primary endpoint of the study was muscle tone, where tolperisone proved to be significantly superior when compared to placebo in attenuating post-stroke spasticity. Interestingly adverse effects occurred less often in the tolperisone group than in the placebo group and caused no need to discontinue the therapy, further proving the preferable tolerability profile of tolperisone [16]. Because of the chiral center next to the carbonyl group in the molecular structure of tolperisone (Figure 2), stereoisomers exist. For medication purposes the racemic mixture is generally used. D-tolperisone is responsible for central skeletal muscle relaxation, while implications were raised about L-tolperisone inducing vasodilation and bronchodilation [178,179]. Fels performed a comparison of tolperisone’s structure to the biologically active conformations of lidocaine and other local anesthetics [178]. The study concluded that, because of the distance and orientation of presumed protein binding structural elements and the similar electrostatic potential pattern, tolperisone, lidocaine, and other local anesthetics could share a protein surface and binding site that can bind all ligands. It is important to also note that, while these drugs may share binding sites because of a similar conformation, the local anesthetics, but not tolperisone, can be superimposed with other, slightly different orientations; therefore, they may reach other binding sites that are unavailable to tolperisone. This could explain the lack of antiarrhythmic potential of the latter compound.

As a summary, tolperisone acts as a multi-target drug; however, its multiple molecular targets call attention to the clinical value of tolperisone in the treatment of NP. Indeed, further studies are needed to elucidate the effect of tolperisone on different chronic pain conditions of different pathological entities and to extend it to human subjects.

## 3. Current Knowledge on the Efficacy of CMRs in Neuropathic Pain Management

The existing literature indicates that the efficacy of CMRs in the management of NP is a subject of ongoing research. Although CMRs show promising results for several painful neuropathic conditions, there is also a significant amount of negative findings and low-quality evidence in the literature. Notably, despite the fact that a large number of painful musculoskeletal and CNS disorders are associated with muscle spasms, the efficacy of CMRs as a class is controversial. While there is evidence that, in certain conditions, the potential benefits of these agents outweigh the risks, it is important to pinpoint these conditions, as their use is limited by the almost universal side effect of sedation or, in some cases, the potential for abuse, such as in case of carisoprodol [31,180,181,182]. As an example, the European Medicines Agency (EMA) conducted a review of the safety and efficacy of tolperisone and decided to restrict its use to the treatment of post-stroke spasticity, due to insufficient evidence that its benefits outweigh the risks in treating other painful spasticities (https://www.ema.europa.eu/en/medicines/human/referrals/tolperisone accessed on 15 December 2025).

While baclofen does show promise in the treatment of TGN [51], a small clinical study published in 1985 did not demonstrate analgesic efficacy in postherpetic neuralgia (PHN) or painful diabetic neuropathy [183]. Notably, within the same cohort, the authors reported a more favorable response among patients with facial PHN, suggesting possible phenotype- or localization-dependent effects [183]. In chemotherapy-induced PN, topical treatment with an organogel containing baclofen, amitriptyline, and ketamine also failed to meet the primary endpoint of the study for symptom improvement [184].

Evidence supporting tizanidine for NP remains inconclusive, largely because adequately powered, placebo-controlled trials are scarce. Its effectiveness has been shown in clinical trials; however, these often represent low-quality studies without a placebo comparator [102].

To the best of our knowledge, despite encouraging preclinical findings, tolperisone has not been evaluated in randomized clinical trials targeting classic NP indications. Similarly, while cyclobenzaprine has demonstrated benefit over placebo in fibromyalgia, placebo-controlled trials in established NP syndromes are missing.

Taken together, the analgesic value of CMRs remains uncertain, both in NP and across their traditional spasm-related applications, where risk–benefit concerns have recently emerged. For repurposing them for NP indications, high-quality clinical trials would be necessary to better characterize their risk–benefit ratio and provide solid evidence for their clinical use. The currently available low-quality evidence also highlight a persistent gap in translation between mechanistic rationale, preclinical efficacy, and clinical outcomes.

Significant efforts have been made at both preclinical and clinical levels to enhance the therapeutic effects while simultaneously reducing adverse effects, including strategies based on combination drugs that target different molecular pathways [185]. This approach has shown promise in managing NP, particularly with the combination of VGSC and VGCC blockers, which has demonstrated efficacy in reducing the NP of rats [142]. Other earlier preclinical studies also supported this combination-based therapy. As mentioned in the former section, higher doses of the pregabalin/carbamazepine combination produced a synergistic antiallodynic effect in the NP model in rats; however, the side effects were not evaluated [145]. In addition, Essmat et al. in 2023 showed that combining tolperisone with pregabalin at low doses provided acute antiallodynic effect in rats with mononeuropathic pain. Also, the combination is devoid of adverse effects related to motor coordination or delay in the gastrointestinal transit of rats [142]. However, these studies lack data regarding pharmacokinetic interactions between the studied drugs. The use of cyclobenzaprine in combination with other medications to manage NP, particularly those that target ion channels, is limited. Although cyclobenzaprine per se or in combination, specifically with drugs for treating NP, is not well-established in the existing literature, further evaluation is necessary regarding its pharmacodynamic and pharmacokinetic interactions with current medications for NP.

Analgesic effect and NP mechanisms are known to be sex-dependent. It is impossible to rule out any sex-related variations in efficacy because most of the preclinical studies assessing CMRs have been carried out in male animals. With respect to tolperisone, two preclinical studies conducted by our research group evaluated its antiallodynic effects in rat models of NP [141,142]. However, these studies have not investigated potential sex differences in the antiallodynic efficacy of tolperisone. Therefore, potential sex-dependent differences in tolperisone efficacy remain unknown. In relation to tizanidine, a study carried out by Rodríguez-Palma et al. 2022 in discussed the sex-specific antiallodynic effects of tizanidine in experimental NP in rats [186]. The authors have shown that increasing doses of tizanidine produced a dose-dependent antiallodynic effect in neuropathic rats and the analgesic efficacy was greater in female than in male animals [186]. The authors have also highlighted the contribution of estradiol to the enhanced antiallodynic effect of tizanidine in female rats. Studies of NP should consider the underlying subject-specific mechanisms, sex, genotype, and other variables such as Nav subtype expression, sensory phenotype among others [125,187,188,189,190,191]. Only with these considerations can we hope to develop safe and effective treatments for NP.

## 4. Future Perspectives and Conclusions

In fact, to date, in various populations with neuropathic conditions, a single drug therapy from currently available medications is often inappropriate in NP as indicated by the high number of patients needed to treat. VGSC blockers, specifically those block Nav subtypes participating in pain modulation, are of future interest. Carbamazepine is a VGSC inhibitor, is one of the first line medications for managing TGN and also presented in the drug list composing lines of medication for the treatment of NP, however its pharmacokinetics interactions and side effect limit its use. This enforces researchers to find selective Nav subtype inhibitors or repurposing drugs acting on these channels. The gathered data support the effectiveness of selective Nav inhibitors in NP. Combining two or more drugs with different mechanisms of action has gained increasing attention in NP management and is applied in the current treatment approaches. CMRs have various chemical structures that are reflected by their multiple mechanisms of action, yet further mechanisms and effects still need to be clarified. Among the elucidated mechanisms, some are also attributed to agents currently used to treat NP including VGSC, VGCC, and spinal serotonin or noradrenergic system modulation. Despite similar pharmacological properties, at the clinical setting, the analgesic effect of CMRs is not utilized yet in NP. The use of preclinical models with high translational relevance (e.g., non-human primates) alongside the rodent pain models may provide a critical step towards clinical feasibility in the future. Based on preclinical results, a combination of CMRs with multiple modes of action related to the alleviation of neuropathic symptoms might offer great promise in overcoming the drawbacks of current treatment approaches. Nevertheless, decreasing muscle spasticity together with the inhibition of pain sensation by combining certain CMRs with drugs belonging to the available treatments for NP might be needed to achieve effective management. In this scenario, the combination of tolperisone with pregabalin can open a future avenue for research NP.

As a limitation of this review, the development of tolerance, receptor desensitization, or channel upregulation may limit the long-term utility of CMRs in the management of NP.

## Figures and Tables

**Table 1:** Comparative data of CMRs and medications for neuropathic pain.

	Compound	Molecular Targets/Effect	Clinical Use	Onset of Action	Side Effects	Clinical Considerations	References
CMRs	Cyclobenzaprine	- 5-HT antagonist - Serotonergic descendant pathway inhibition - Anticholinergic effect	Adjunctive treatment of acute painful musculoskeletal conditions associated with muscle spasm	1 h (single dose)	Drowsiness, dry mouth, blurred vision, increased intraocular pressure, urinary retention, constipation, confusion, dyspepsia, serotonin syndrome, TCA-like toxicity	Cannot be used with monoamine-oxidase inhibitors. Withdrawal symptoms may arise upon prompt discontinuation.	[31,32,33,34,35,36]
	Tizanidine	α_2_-AR agonist	Used in a wide range of conditions involving muscle spasticity	1–2 h (single dose)	Drowsiness, vertigo, hypotension, dry mouth, asthenia, slurred speech	The common side effect of sedation taken together with the short half-life and the need for multiple daily administrations may limit its use.	[37,38,39,40,41]
	Tolperisone	Inhibition of VGSCs and VGCCs	Painful muscle spasms, post-stroke spasticity, and other conditions involving muscle spasticity	1 h (single dose), may need a longer treatment period for maximal benefit	Well-tolerated, minor adverse effects include dry mouth, nausea, sleep disturbances	Lacks the common side effect of sedation seen with all other CMRs.	[17,18,42,43,44,45,46,47]
	Baclofen	GABA_B_R agonist	Management of muscle spasticity associated with spinal lesions or multiple sclerosis	Highly variable (hours-weeks)	Drowsiness, confusion, headache, nausea, hypotension, hypothermia	Dangerous withdrawal symptoms (including seizures) may arise upon prompt discontinuation. May be useful in the treatment of trigeminal neuralgia.	[31,48,49,50,51]
Medications for NP	Gabapentin/Pregabalin	Inhibition of VGCCs containing α_2_δ_1_ subunit, A1 receptor agonism	1st-line recommendation in NP, best established in PN	Maximum plasma concentration is reached after 3.2 h, but analgesic onset against NP is slow- weeks	Well-tolerated, drowsiness, dizziness, peripheral edema, weight gain	Highly variable NNT for 50% pain reduction in different NP syndromes. Using a symptom rather than syndrome-based approach, NNT of gabapentin is between 7.2 (95% CI 5.9–9.1) and 14 according to different sources.	[25,52,53,54,55,56,57]
	Duloxetine	SNRI, weak inhibition of dopamine reuptake	1st-line recommendation in NP	1–2 weeks, may take up to 4–6 weeks for maximal benefit	Nausea, dry mouth, dizziness, somnolence or insomnia, constipation, increased blood pressure, sweating	Best established in diabetic neuropathy, where NNT for 50% pain reduction is 5. For mixed-syndromes NP 6.4 (95% CI 5.2–8.4). Caution is needed in patients with hepatic impairment or uncontrolled hypertension.	[52,58,59]
	Amitryptiline (TCAs)	- Inhibition of neuronal 5HT and NE reuptake. - Antagonist effects on histamine receptor 1, α_1_-AR, muscarinic receptors, 5-HT2_A_ receptor, NMDAR. - Activated VGSC inhibition.	1st-line recommendation in NP	1–2 weeks, may take up to 4–6 weeks for maximal benefit	Sedation, dry mouth, constipation, urinary retention, orthostatic hypotension, weight gain, cardiac conduction abnormalities	NNT for 50% pain reduction (mixed NP syndromes) is 3.6 (95% CI 3.0–4.4), which includes peripheral nerve injury, radiculopathy and CPSP, among others. While the NNT is the lowest, the tolerability is also poor, owning to several off-target CNS effects.	[25,52,57,60]
	Lidocaine	- VGSC inhibition	2nd-line recommendation in NP	Min to hours (topical)	Local skin irritation (topical), local anesthetic systemic toxicity (LAST, incl. paresthesias, cardiac arrythmias, seizures, coma) at high systemic doses	Most commonly used as topical 5% patch for localized PN pain; favorable safety profile compared to systemic agents. The NNT for 50% pain reduction is undetermined owning to low-quality evidence.	[25,52,61,62]
	Tramadol	- μ-, δ-, and κ-opioid receptor agonism, serotonin (mainly the (+)-enantiomer) and norepinephrine (mainly the (−)-enantiomer) reuptake inhibition	2nd-line recommendation in NP	Approx. 1 h after oral administration	Nausea, dizziness, constipation, sedation, risk of seizures, serotonin syndrome	Based on moderate-quality evidence from short-term trials, the NNT for 50% pain reduction in NP (mixed-syndromes) is 4.7 (95% CI 3.6–6.7). Dependence potential and interaction risk limit its use.	[52,63,64,65]
	Carbamazepine	- VGS and VGC channels inhibition, A1 receptor antagonism	1st-line recommendation in lancinating and neuritic pain syndromes (e.g., TGN or glossopharyngeal neuralgia)	Days to 1–2 weeks for NP relief	Sedation, vertigo, ataxia, blurred vision, rash, aplastic anemia, hyponatremia	High incidence of serious unwanted effects necessitates strict monitoring regime for those on carbamazepine therapy.	[52,66]

A comparison of the pharmacological profiles, clinical applications, onset of action, adverse effects, and significant considerations of selected CMRs and first and second-line therapies for NP management. PN: peripheral neuropathy.

**Table 2:** Nav1 subtypes and their ligands with analgesic effect.

Drug	Nav Subtype	Subject	Pain Model	Findings	**Reference**
QLS-81	Nav1.7	Mice	SNI-induced NP	- inhibits Nav1.7 current—more potent than rafinamide - causes a hyperpolarizing shift of fast and slow inactivation of Nav1.7 and slows down inactivation recovery - inhibits native Nav current and suppresses neuronal firing in mouse DRG neurons - alleviates NP - no significant effect on ECG and spontaneous locomotor activity in mice	[130]
E0199	Nav1.7, Nav1.8, Nav1.9 (Kv7.2/7.3, Kv7.2, and Kv7.5)	Rats (DRG) Mice (pain behavior studies)	CCI	- inhibits Nav1.7, Nav1.8, and Nav1.9 (particularly low IC_50_ for Nav1.9) - increases Kv7.2/7.3, Kv7.2, and Kv7.5 channels’ activity - reduces the excitability of DRG neurons - alleviates thermal, mechanical, and cold hypersensitivity in CCI hypersensitivity in mice at low doses - therapeutic effects persisted even after treatment discontinuation	[131]
A-803467	Nav1.8 (100-fold selective than Nav1.2, 1.3, 1.5 and 1.7) [human]	Rats (DRG, pain models) Human (recombinant Nav channels) Mice (abdominal constriction assay)	SNL, CCI sciatic nerve injury, capsaicin-induced allodynia, vincristine model of CINP (+ rat DRG firing patterns, recombinant human Nav-s)	- blocks Nav1.8 currents in rat DRG and HEK-293 cells expressing recombinant human Nav1.8 channels - reduces evoked and spontaneous DRG neuronal action potentials - attenuates CCI evoked mechanical allodynia and capsaicin-induced secondary mechanical allodynia - attenuates thermal hyperalgesia (cold allodynia in CCI model) - no effect on vincristine model of chemotherapy-induced mechanical allodynia - no effect at the 2 h post-op timestamp in the skin-incision model of postoperative pain (had a small, but significant effect 24 h post-op)	[132]
A-803467	Nav1.8	Rats	SNL	- Iv. ↓ spontaneously and mechanically evoked WDR neuronal firing in SNL rats; ↓ transiently evoked activity in uninjured rats, but spontaneous firing remained intact - intraspinal decreased both evoked and spontaneous WDR neuronal firing - injection into L4 DRG or into the hindpaw receptive field attenuated evoked but not spontaneous firing - systemic (Iv.) effects in SNL rats were not altered by systemic pretreatment with resiniferatoxin—TRPV1 agonist	[133]
LTGO-33	Nav1.8	Human and rat Nav Rat, mouse, beagle dog, cynomolgus monkey, human DRG	-	- shows species specificity for primate Nav1.8 over dog and rodent Nav1.8 and inhibited action potential firing in human DRG neurons - - also blocks Nav1.8 variants associated with human pain disorders	[134]
QLS-278	Nav1.7, (TTX-S Nav1.4, TTX-R Nav1.5, and TTX-R Nav1.8 with lower potency)	Mice, cell line	SNI model of NP	- ↓ native TTX-S Nav currents and action potential firing in DRG neurons - dose-dependently alleviated SNI-NP - no significant effect on locomotion	[135]
19h	Nav1.7	Mice	SNI	- inhibited Nav1.7 currents in a dose-dependent manner and 12-fold > ralfinamide - dose-dependently increased pain threshold in the SNI model - no sedation	[136]
Suzetrigine	Nav1.8	Phase 3 clinical trial	Moderate-to-severe acute pain after abdominoplasty or bunionectomy	- shows analgesia - produced a 2-point or greater decrease in the numeric pain rating scale score more rapidly than placebo - at 48 h, showed similar analgesia as hydrocodonebitartrate/acetaminophen - well tolerated, most adverse effects being mild to moderate	[128]
Suzetrigine	Nav1.8	Phase 3 clinical trial	Pain after surgical procedures or non-surgical pain of new origin	- its analgesic effectiveness was rated as good, very good, or excellent on a patient global assessment by 83.2% of participants	[137]
Vixotrigine	Nav1.7	Phase 2A clinical trial	TGN	- no significant difference in treatment failure between vixotrigine and placebo - significant difference in time to treatment failure, - paroxysm number, average daily pain score, assessment of overall function and quality of life compared to placebo - well tolerated, most common adverse event was - headaches	[138]
Vixotrigine	Nav1.7	Mice	Model of acute postsurgical pain (incision of the plantar skin and underlying muscle of the hind paw)	- po. administration reduced mechanical allodynia	[139]
Raxatrigine	Nav (non-selective)	Mice	OD1 toxin model (mostly selective Nav1.7 activator)	- ip. administration attenuated spontaneous pain - behaviors (highest dose had sedative adverse - effects) - ineffective as a local intraplantar injection	[140]

↓: decrease.

## Data Availability

No new data were created or analyzed in this study.

## Abbreviations

The following abbreviations are used in this manuscript:

AR	Adrenoceptor
CCI	Chronic constriction injury
CINP	Chemotherapy-induced neuropathic pain
CMR	Centrally acting skeletal muscle relaxant
CNS	Central nervous system
DRG	Dorsal root ganglion
ECG	Electrocardiogram
GABA_B_R	gamma-aminobutyric acid B receptor
GPCR	G-protein coupled receptor
HERG	Human ether-a-go-go-related gene
h	Hour
HVA	High-voltage-activated
IC_50_	Half-maximal inhibitory concentration
IKr	Rapid delayed rectifier potassium current
Ip.	Intraperitoneal
Iv.	Intravenous
LVA	Low-voltage-activated
MAPD90	Monophasic action potential duration at 90% repolarization
NMDAR	N-methyl-D-aspartate-receptor
NP	Neuropathic pain
NSAID	Non-steroidal anti-inflammatory drug
PHN	postherpetic neuralgia
PN	Peripheral neuropathy
Po.	Per os
SCI	Spinal cord injury
SNI	Spared nerve injury
SNL	Spinal nerve ligation
TCA	Tricyclic antidepressant
TGN	Trigeminal neuralgia
TRPV1	Transient Receptor Potential Vanilloid 1
TTX-R	Tetrodotoxin-resistant
TTX-S	Tetrodotoxin-sensitive
VGCC	Voltage-gated calcium channel
VGSC	Voltage-gated sodium channel
WDR	Wide dynamic range
